# The reinforcement sensitivity theory affects questionnaire (RST-AQ). A validation study of a new scale targeting affects related to anxiety, approach motivation and fear

**DOI:** 10.1007/s12144-023-04623-z

**Published:** 2023-05-09

**Authors:** Vittoria Franchina, Johannes Klackl, Eva Jonas

**Affiliations:** Department of Psychology, University of Salzburg, Salzburg, Austria

**Keywords:** Reinforcement sensitivity theory (RST), Anxiety approach fear, Affects, Self-report measure, Validation

## Abstract

This paper presents the RST-AQ, a 22-item scale to measure the affective states related to the three motivational systems postulated by Reinforcement Sensitivity theory (RST-AQ): the Behavioral approach system (BAS), Behavioral inhibition system (BIS), and the Fight-Flight-Freeze system (FFFS). The three subscales are internally consistent. Results show an overall support for construct validity of our RST-AQ measure. The correlations of the RST-AQ subscales with other measures demonstrate a good convergent and divergent validity with regard to the subscales of BAS and BIS. The RTS-AQ Scale provides researcher with the first instrument to measures the affective states of the RST theory.

## Introduction

According to Reinforcement sensitivity theory (RST) there are three systems underlying responses to motivationally significant stimuli. The behavioral approach system (BAS; Corr & Cooper, 2016) mediates behavior toward positive/rewarding/appetitive stimuli, and it is believed to be associated with approach-related affects, such as enthusiasm, interest and joy. The Fight-Flight-Freeze system (FFFS; Corr & Cooper, 2016) underlies behavior away from negative/punishing/repulsive stimuli, and is accompanied by fearful affects. The Behavioral Inhibition system (BIS; Corr & Cooper, 2016) regulates conflicts between motivational tendencies, and is associated with anxiety. For example, the same sight of a dog can elicit quite different motivational, behavioral and emotional responses in different people: some might experience positive emotions and want to approach the dog. Others might experience fear and, hence, want to run away from the animal; yet others might experience anxiety and wonder whether it is safe or not to approach the animal. According to RST, these different ways of responding are accompanied by predominant activation of the BAS, FFFS and BIS, respectively. RST explicitly argues that individual differences in the strength or sensitivity of the three systems are a major source of personality differences (Corr & Cooper, 2016). This idea has given rise to a number of personality scales that measure temporally stable tendencies of people with which they activate their BIS, BAS and FFFS (Carver & White, 1994; Corr & Cooper, 2016).

Reinforcement sensitivity theory (RST) is not only a central theory in personality psychology; it also provides the basis of social psychological accounts of psychological conflict (McGregor et al., 2010; Nash et al., 2011; Harmon-Jones et al., 2016). These accounts commonly assume that activity levels can change dynamically in response to specific situations. These dynamic activations cannot be meaningfully measured with personality scales because such scales measure traits that are relatively stable over time. Instead, it is common practice to assess the momentary (changes in) activation of BIS and BAS with various ways; such as electroencephalography (Harmon-Jones et al., 2016; Nash et al., 2011; Agroskin et al., 2016) or the line bisection task (e.g., Bowers & Heilman, 1980; Milner et al., 1992; Fischer, 1994; Guinote, 2017). These approaches are based on the assumption that higher activity of the left hemisphere of the brain is associated with approach motivation. However, the line bisection task has been repeatedly shown to have little to no validity as indicator of approach motivation (Lee et al., 2004; Leggett et al., 2016; Hatin &Tottenham, 2016; Pugnaghi et al., 2018; Pugnaghi et al., 2019) and electroencephalography is difficult, expensive and time-consuming. Therefore, measures of affects would provide an interesting and economically sensible window into dynamic activation of BIS, BAS and FFFS. Researchers (e.g., Carver, & White, 1994; Greenaway et al., 2015; Bacon et al., 2017; Wytykowska et al., 2021; Wilson et al., 2021) have extensively shown that affects are indicators of the activation of BIS, or BAS or FFFS. In light of this, it is surprising that, as far as we know, there is no comprehensive, emotion-based instrument to assess the strength of BIS, BAS, and FFFS. Although there are already many existing traits based instruments (e.g., Torrubia et al., 2001; Corr & Cooper, 2016; Espinoza Oyarce, et al., 2021), there are no validated instruments to assess the affective aspects of the RST systems.

Following this line of thoughts an affect-based measure to assess the strength of BIS, BAS, and FFFS is extremely needed. That is because many before us have investigated affects in the context of the reinforcement sensitivity theory, adapting various already existing self-report measures (e.g., Merchàn-Clavellino et al., 2019; Bacon & Corr, 2017; Heponiemi et al., 2003) Nevertheless, these scales were validated to assess clinical affective conditions, but not validated for the purpose of tracking dynamic changes in affects based on the reinforcement sensitivity theory. For example, Hundt et al. (2007) measured BIS affects via the Anxious Arousal scale; The Anhedonic Depression scale, a General Distress Depression scale and a General Distress Anxiety scale. Indeed, measuring variables with adapted instruments presents limitations, as it is unsure if one is really assessing the theoretical construct intended to be measured. The purpose of the present paper is to provide researchers investigating fearful, anxious and approach-related affects based on RS theory with an instrument validated just for this purpose.

This research project is relevant for at least three reasons. First, as mentioned above, it serves scientists studying BIS, BAS and FFFS related affects with a short and easy to fill in self-report measure. Second, the project is valuable as the insights garnered from this research contributes to extend the current knowledge on these three affects in relation to three motivational systems underlying behaviors. Third, insights can be used for future studies aiming to further disentangle these three affects.

## Theoretical framework

According to reinforcement sensitivity theory (RST; McNaughton & Gray, 2000; McNaughton & Corr, 2004, 2008; Corr & McNaughton, 2012; Espinoza Oyarce, et al., 2021), three motivational systems underlie behavior: the behavioral inhibition system (BIS), the behavioral approach system (BAS) and the fight-flight-freeze system (FFFS). Already in the 1980s, individual differences in sensitivity of these neurological systems have been related to different personality dispositions in reacting to the environmental stimuli (e.g., Fowles, 1987; Carver & White, 1994). Moreover, each one of these motivational systems is thought to produce typical affective experiences related to interest and joy in the case of BAS, or anxious and fearful affect in the case of BIS and FFFS, respectively (e.g.,Carver & White, 1994; Greenaway et al., 2015; Wytykowska et al., 2021; Moncel, et al., 2023). As mentioned before, the BIS needs to be distinguished from the FFFS system (Corr & Cooper, 2016). The FFFS is activated by unambiguously negative, repulsive, threatening stimuli. The BIS is activated by conflict, which might arise, for example, from ambiguous stimuli that co-activate two conflicting behavioral tendencies (Carver & White, 1994), i.e., tendencies to approach and actively avoid something. Thus, BIS activation often arises when a stimulus or situation is not clearly rewarding or threatening, or when it is both, attractive and repulsive. This results in the inhibition of behavior and the engagement in risk evaluation processes with the final purpose of deciding which behavior to undertake (Corr & Cooper, 2016). Researchers report that FFFS elicits *fear* and terror, whilst BIS *anxiety* (e.g., Corr & Cooper, 2016).

In what follows, these affective constructs are discussed in two steps. In a first step, each one of the three systems will be discussed more in detail in its own dispositional and especially affective aspects. In a second step, an overview of the current understanding of these affects is provided.

### Approach-related affects. “Let’s go for it!”

The BAS regulates desire-related motivation (appetitive motivation; e.g., Franken, 2002; Carver & White, 1994). The engagement in goal directed behavior and the experience of positive feelings in hope of obtaining reward are indicators of BAS activation (Corr & Cooper, 2016). However, each individual might have different levels of the strength of that system (also referred to as BAS sensitivity; Carver & White, 1994), which might explain the individual differences in approach directed behaviors in the presence of reward cues. For example, higher BAS sensitivity is related to addictive behaviors (e.g., Franken, 2002; Kidd & Loxton, 2021) and bipolar disorders (e.g., Alloy et al., 2009). On the contrary, low BAS sensitivity predicts depression (McFarland et al., 2006).

Besides exploring how BAS sensitivity relates to clinical disorders (e.g., Chat et al., 2022), researchers (e.g., Greenaway et al., 2015; Harmon-Jones et al., 2016) have being exploring approach-related affects elicited by BAS activation. As already mentioned, these affects are indicators of the systems’ activation levels. The BAS can be activated in response to various stimuli, many of which are associated with goals. Moreover, while approaching a given goal, the BAS engages a number of relatively separate, although overlapping, processes (Corr, 2004) associated with different affective states that serve to maintain motivation across space/time (Corr & Cooper, 2016). At the early stages of these processes we can differentiate between ‘reward interest’ and ‘goal-drive persistence,’ whilst ‘reward responsivity’ and ‘impulsivity’ characterize the late stages of goal pursuit. Reward Interest concerns the initial motivation to seek out potentially rewarding places, objects, activities or people. Goal drive persistence relates to actively pursuing a desired goal after having identified it, especially when reward is not immediately available. Reward responsivity relates to excitement at doing things well, especially to rewarding stimuli associated with fulfilling sub-goal procedures. Lastly, Impulsivity relates to behaviors closer to the final biological reinforcer, which no longer entails planning or restraint of behavior. Similarly, Carver and White (1994) have described the affects experienced in the early stages of approaching a reward as “anticipatory pleasure” or “hope” and “high pleasure” or “joy” those at the latest stages of the overall process.

In line with these results (Carver & White, 1994; Corr & Cooper, 2016), Egloff et al. (2003) investigated how a number of positive affect subtypes changed during approach task, and proposed to differentiate these into Interest, Activation and Joy. *Interest* was high and stable throughout the study. *Activation* increased during the task and decreased afterwards; *joy* increased after the task (Egloff et al., 2003). Therefore, as *interest* seems to be a more stable than the other two, which change according to the process’ phase (Egloff, et al., 2003), it is likely to find an higher correlation between *activation* and *interest* as well as *joy* and *interest*. Although all the three affects should correlate positively and strongly with each other.

Moreover, it is likely to find an higher correlation between *activation* and the dimensions of the early stages of the process, *joy* and the late stages, and *interest* with both early and late.

Among the above mentioned affects, anger has also been claimed to be an approach-related affect (e.g., Cooper et al., 2017). Findings (e.g., Carver, 2004; Cooper et al., 2008; Khoshfetrat et al., 2022) have shown that high BAS sensitivity predicted expressing anger outwardly or even acting on it. Gomez and colleagues argued that this is because high trait BAS people’s anger results from the frustration of not achieving a goal. Therefore, expressing anger outwardly or behaving aggressively would represent an approach-oriented solution (Cooper et al., 2008). Nevertheless, an aggressive behavioral response might result from BAS as well as FFFS activation (Cooper et al, 2008). That is because such behavior might be a goal approach or predatory aggression, thus BAS oriented, or a defensive fight, thus FFFS oriented. Therefore, this research meant to explore whether anger (measured by STAXI self-report; Vagg & Spielberger, 1979) might be an indicator of the BAS activation system.

### Fear-related affects. “Get me out of this place!”

The FFFS system mediates active avoidance of threats. Generally speaking, threatening stimuli that can simply be avoided elicit defensive behaviors of *Flight* and *Active Avoidance* (according to defensive distance or perceived threat); whilst those that cannot be easily avoided elicit behaviors of *Fight* and *Freeze* (Corr & Cooper, 2016)*.* As with the BAS, some individuals have a more sensitive FFFS. For example, flight responses have been associated with panic disorders (e.g., Blanchard et al., 2001).

The FFFS is believed to mediate the emotion of fear, not anxiety (e.g., Corr & Cooper, 2016). Fear and anxiety have been demonstrated to be distinct entities (Perusini & Fanselow, 2015): a state of fear is sensitive to panicolytic drugs but not to those that are only anxiolytic and work with anxiety (e.g., McNaughton & Corr, 2004). Interestingly, these different drugs have specific effects on behaviors, which allowed researchers to distinguish the behavioral correlates of these two affects: fear operates when there is an active avoidance such as for example leaving a dangerous context; anxiety operates when for example cautiously entering a situation (approach behaviours that engages risk assessment) or when avoiding it (passive avoidance; Gray, 1977). Furthermore, fear is an automatic affective response to aversive stimuli; it is relatively inaccessible to cognitive control and dependent on a neural circuitry that includes the amygdala (Öhman & Mineka, 2001). Whereas actual threat elicits fear, potential threat (that is, the anticipation of aversive contexts or consequences) elicits anxiety (Crocq, 2017). Moreover, anxiety is accompanied by increased arousal, expectancy, autonomic and neuroendocrine activation as well as, of course, behavioral patterns. These adaptations allow us to cope with an adverse or unpredicted situation (e.g., Kaynak et al., 2022; Steimer, 2022). Following this line of thoughts, it is comprehensible why researchers (e.g., Öhman & Mineka, 2001; Corr & Cooper, 2016; Sylvers et al., 2011) doubt that it is possible to distinguish between fear and anxiety on the basis of affective self-report measures. For them, it is likely that via self-report we measure anxiety-related affect associated with the cognitive anticipation of a threat; whereas fear (as an immediate reaction to a present threat) is more easily measured via physiological measures (e.g., Castro-Toledo et al., 2017).

### Anxious-related affects. “Watch out for danger!”

The BIS is responsible for the resolution of goal conflict in general (e.g., between BAS-approach and FFFS-avoidance; McNaughton & Corr, 2004). Activation of the BIS entails the inhibition of conflicting behaviors, the engagement of risk assessment processes, and the scanning of memory and the environment to help resolve concurrent conflict. Conflicts can be elicited by contexts including both reward and threat (i.e., both the BAS and the FFFS have been activated; Corr 2004). If reward outweighs threat, the BIS will resolve the conflict by engaging the BAS and inhibiting the FFFS, resulting in approach. If threat outweighs reward, the BIS will further activate the FFFS and inhibit the BAS, resulting in avoidance (Bijttebier et al., 2009). However, conflicts are not restricted to approach–avoidance conflicts: approach–approach conflicts and avoidance–avoidance conflicts can also occur (Bijttebier et al., 2009).

As mentioned, FFFS mediates *fear* and terror, whilst BIS *anxiety* (e.g., Heidari & Nemattavousi, 2021; Corr and Cooper, 2016). However, besides the motivational and behavioral distinction, the differentiation between these affects is still understudied. For this reason, it is fundamentally important to provide researchers with a psychometric measure to differentiate fear and anxiety, in order to better understand and research these affects. This is what Corr and Cooper pointed out in their paper developing and validating the Reinforcement and Sensitivity Theory of Personality Questionnaire (Corr & Cooper, 2016), highlighting this as a significant obstacle to research progress. Nevertheless, as far as we know, this research need has not been addressed yet.

### Overview of the current understanding of anxious, fearful and approach-related affects

Individual differences in proneness to experience affects are related to different dispositional sensitivity in BIS, BAS and FFFS motivational systems (e.g., Baumann et al., 2007). Findings (e.g., Gross, 2013) suggest that differences in individual dispositional BIS, BAS and FFFS are associated not only with differences in the experience of affects but also in emotional regulation processes. Studies (e.g., Tull et al., 2010; Baumann et al., 2007) reveal significant associations between the dimensions of BIS, BAS, FFFS and emotional regulation. For example, BIS and FFFS are positively associated with difficulties in emotional regulations, whereas certain BAS dimensions such as Drive and Fun seeking are associated with adaptive emotional regulations (Tull et al., 2010). Emotional self-regulatory skills regard, among other things, the speed of entering and leaving an affective state, as well as the capability of self-motivation (Baumann et al., 2007). Furthermore, recent results (Merchàn-Clavellino et al., 2019) show the influence of BIS and BAS motivational systems on the experience of affects, and that this relationship is moderated by emotional intelligence.

Generally speaking, higher BAS and low BIS/FFFS-related affects might lead to greater job satisfaction (Moè et al., 2010), better choices of more effective learning strategies (De Beni & Moè, 2003), different reactions to gender sterotypes (Moè et al., 2021). On the contrary, higher BIS levels are related to depressive symptoms (e.g., Bryan et al., 2022; Wu et al., 2021; Li et al., 2015) and the use of maladaptive emotional regulation strategies (Bijttebier et al., 2009).

Although greater BAS sensitivity appears more often related to positive outcomes (e.g., Hu et al., 2022; Pulido et al., 2023), this is not always the case. For example, BAS impulsivity shows negative associations with self-control (Bacon & Corr, 2017). At the same time, it is likely that exercising self-control increases approach motivation. Also, BIS might be a component of self-control (Schmeichel et al., 2010). In line with these thoughts, because of the complex relationships between these three constructs (e.g., Kajdzik & Moroń, 2023; Strand, 2023, Włodarska et al., 2021), it is important to assess BIS, BAS and FFFS related affects through a combined measure of the three dimensions.

## Aims and hypotheses

The first aim of our paper is to provide just such an instrument. To measure these three kinds of affects, different already existing affective self-report scales are used and combined in a newly developed affect self-report scale named the Reinforcement sensitivity theory affect questionnaire (RST-AQ). Secondary goal is to test the validity of the RST-AQ measure.

In order to test the validity of RST-AQ, the scale was hypothesized to show a satisfactory internal consistency, convergent validity (i.e., our BIS, BAS and FFFS subscales should be positively, significantly and at least moderately correlated with themselves and other affect scales measuring the same constructs) and divergent validity (i.e., our BIS and BAS subscales should correlate more strongly with measures of BIS and BAS traits, respectively, than with measures of a similar but different construct such as pessimism and optimism; Glaesmer et al., 2008).

Moreover, BAS, BIS and FFFS subscales were predicted to correlate positively, significantly and at least moderately with their matching counterparts, while correlating much lower with their non-matching counterparts. For example, our measure of BAS related-affects should correlate more strongly with measure of BAS trait, than with measures of BIS and FFFS trait.

## Method and materials

### Development of scale

Initially, quality criteria were established for assessing the adequacy of our RST-AQ.

First, the scale should assess the affects related to the activation of BIS, FFFS and BAS systems, as identified by the Reinforcement Sensitivity theory of Corr and Cooper (2016).

Second, the scale should have good psychometric properties in terms of internal consistency and convergent validity. Moreover, the scale should show some evidence for divergent validity.

In order to create a scale coherent with the first quality criterion, a pool of 24 items was developed. These items were selected following a deductive method (Boateng et al., 2018) for items generation: through literature review on affects and evaluation of existing measures (e.g.,Greenaway et al., 2015; Harmon-Jones, et al., 2016; Maack et al., 2015; Watson & Clark, 1999).

A first pool of items was generated. An iterative process of evaluation was then undertaken by researchers with expertise in the field, until consensus was reached on face validity. This resulted in a final pool of 24 items including 9 items from the PANAS-X (Watson & Clark, 1999) Positive Affect subscale for our BAS subscale; six items from the PANAS-X Fear subscale and two face valid items (worried and inhibited) for our BIS subscale; and five items from the FFFQ subscale (Maack et al.,; 2015). The items were scored on a 7-point Likert scale (from 0 = does not apply to me at all to 7 = does apply to me completely).

### Sample

288 German-speaking participants (170 males; 143 females; *M* = 37.22 years; *SD* = 11.71) were recruited through the crowdsourcing platform Clickworker. The online survey was administered via LimeSurvey. The sample size was determined a-priori using an online calculator (https://www.danielsoper.com/statcalc/calculator.aspx?id=89). The only criterion for data exclusion from all analyses was failing the reading check. This was a question included at the end of the survey that could only be answered correctly if participants were reading the instructions carefully. As we anticipated that 20% of participants would fail the reading check, we initially recruited 346 participants (20% more participants than needed). Our prediction was confirmed. The study was preregistered (https://osf.io/jqwft/).

### Procedure

Participants were asked to fill out a number of scales that measure both trait and state aspects of behavioural approach, behavioural inhibition and fight-flight-freeze system sensitivity (see Instruments Section). Also, they were presented with an instrument adopted to assess optimism and pessimism (Glaesmer et al., 2012). Lastly, they were asked about demographics (nationality, employment status, education level). As financial incentive, they received 2.25 € for participation.

### Instruments

Once again, the Reinforcement sensitivity theory affect questionnaire is a newly developed measure of anxious, approach related and fearful affects, and its final version is composed of 22 items (see Appendix).

In what follows, the existing scales used in our study are reported, in the same order these were proposed to participants:

High Approach Scale (Greenaway et al., 2015). The scale measures feelings of high approach. Participants respond to the statement ‘right now I feel’, reporting the extent to which they feel energized, powerful, capable, and competitive on a scale from 1 = not at all to 7 = very much. The measure shows a satisfactory reliability level α = 0.78.

The BIS/BAS Scale (Carver & White, 1994). The measure includes one BIS subscale and three BAS-related subscales: reward responsiveness, drive, and fun seeking. Reward responsiveness’ items focuses on positive responses to the occurrence of anticipation of reward. Drive’s items regard the persistency in pursuing a desired goal. Fun seeking’s items focuses on both desire for new rewards and the willingness to approach a potential reward in the instant it arises. The scale consists of 20 items (e.g., BIS item: if I think something unpleasant is going to happen, I usually get pretty “worked up”; BAS Reward Responsiveness item: when I get something I want, I feel excited and energized) evaluated on a 4-point Likert scale with 1 indicating strong agreement and 4 indicating strong disagreement. The scale shows a satisfactory construct validity and internal consistency with reliabilities of α = 0.74 for BIS, α = 0.73 for BAS Reward Responsiveness, α = 0.76 for BAS Drive, α = 0.66 for BAS Fun Seeking.

State-Trait Anger Expression Inventory (STAXI; Spielberger et al., 1988). The scale provides a self-reported measure of the experience, expression and control of anger, including 44 items that score on a 4-point Likert scale. For our study we included only 10 items reflecting the experience of anger (e.g., I get angry quickly).

Reinforcement Sensitivity Theory Personality Questionnaire (RST-PQ; Corr & Cooper, 2016). RST-PQ includes 84 items (e.g., BIS item: I feel sad when I suffer even minor setbacks; BAS Reward Interest item: I get carried away by new projects; FFFS item: I sometimes wake up in a state of terror) rated on a 4-point Likert-style scale with the options 1 = ‚not at all’, 2 = ‘slightly’, 3 = ‘moderately’, 4 = ‘highly’. Psychometric properties of the RST-PQ were acceptable. Cronbach’s alpha values for FFFS, BIS, BAS Reward Interest, BAS Goal Drive Persistence, BAS Reward Reactivity, BAS Impulsivity were 0.78, 0.93, 0.75, 0.86, 0.78, 0.74.

The Fight, Flight, Freeze Questionnaire (FFQ; Maack et al., 2015). FFQ measures the construct of fear. It includes 21 items (e.g., petrified), evaluated on a 5-point Likert scale. The measure is reliable (α = 0.92).

Life Orientation Test Revised (LOT-R; Glaesmer, et al., 2012). The instrument is adopted to assess optimism and pessimism. It consists in 10 items, rated on a 5-point Likert scale, from strongly agree to strongly disagree. Cronbach’s alpha coefficients of reliability for optimism, pessimism and total score were 0.70, 0.74, and 0.68.

The Descrete Emotions Questionnaire (DEQ; Harmon-Jones, et al., 2016). DEQ is a short measure of basic emotions, including 8 subscales. Each measured by 4 items. For our study, we used the two subscales Anxiety and Fear (e.g., Anxiety items: worry, anxiety, dread, nervous; Fear items: terror, scared, fear, panic). Cronbach’s alpha values for Anxiety and Fear are 0.90 and 0.92.

### Data analyses

In the first step, the internal consistency of the RST-AQ subscales (BIS, BAS, and FFFS) was investigated. Then, the factorial structure of the pool of 24 affects items was examined with an exploratory factor analysis using principal axis factoring and the Varimax with the Kaiser Normalization rotation method, using IBM SPSS Statistics from Window, version 27. In the second step, convergent and divergent validity were investigated by correlating the BIS, BAS, and FFFS subscales of the RST-AQ with (1) other measures of BIS, BAS and F FFS affect and (2) trait measures of BIS, BAS and FFFS. Lastly, correlations between the three subdimensions of BAS (Interest, Activation and Joy) were performed in order to investigate how these dimensions are related to each other.

## Results and discussion

### RST-AQ. Internal consistency and factorial validity

#### Internal consistency

The scale shows a good reliability. Cronbach’s alpha of BAS, BIS and FFFS is 0.88, 0.91, 0.89.

#### Exploratory factor analysis

Examination of the Eigenvalues and scree plot suggested a 3 factors solution. The first factor had an Eigenvalue of 9.35 and explained 38.95% of variance. The second and third factors had Eigenvalues of 3.74 and 1.76. They explained the 15.573% and 7.330% of the variance, respectively. The items that loaded positively on these three factors are displayed in Table 1. Their loadings ranged from 0.539 to 0.783. These values are regarded as sufficient to indicate that the three factors are reliable (Stevens, 2009). We excluded from the final list of items two items: *endangered* and *attentive* because their loadings’ difference on two different factors was less than 0.20. Cross-loadings of items can be observed for the FFFS factor. However, one suggested solution to the problem is to assign the items to the factor that it loads most highly on (Field, 2009; Henson & Roberts, 2006) which in our case would be FFFS. Moreover, Pett et al. (2003) suggest a cutoff score of 0.40 above which loadings are considered important. The BAS, BIS and FFFS subscales were highly internally consistent (see Table 1).

**Table 1:** Exploratory factor analysis

		Factor		
	Subscale	BIS	BAS	FFFS
Active	BAS (α = 0.88)		0.632	
Alert			0.539	
Attentive			0.537	-0.411
Determined			0.630	
Enthusiastic			0.746	
Excited			0.698	
Inspired			0.701	
Interested			0.616	-0.383
Proud			0.664	
strong			0.677	
Afraid	BIS (α = 0.91)	0.723		
Scared		0.705		0.353
Frightened		0.650		0.449
Nervous		0.778		
Jittery		0.679		
Shaky		0.783		
Worried		0.689		
Inhibited		0.649		0.311
Horrified	FFFS (α = 0.89)	0.344		0.704
Terrified				0.772
Petrified		0.376		0.706
Startled		0.357		0.744
Shocked		0.328		0.733
Endangered		0.415		0.551

Extraction Method: Principal Axis factoring. Rotation Method: Varimax with Kaiser Normalization. Values lower than ± .30 are not displayed

#### Confirmatory factor analysis

CFA was performed to test the three-factor model. The goodness of fit of the model was assessed considering the following fit indices: the overall model chi-square (χ^2^), CFI, root mean squared error of approximation (RMSEA), and standardized root mean square residual (SRMR). CFI values of 0.90 and above, and RMSEA and SRMR values of less than 0.08 are considered an acceptable fit (Hu & Bentler, 1999; Kline, 2005). Using these criteria for evaluation, the initial model fit for the three-factor model meet a common fit standard ( χ^2^ (206, *n* = 288) = 628.00, *p* < 0.001, CFI = 0.90, RMSEA = 0.08, SRMR = 0.07).

##### Convergent validity

Data shown in Tables 2 and 3 provide the correlations between our BAS, BIS and FFFS subscales with themselves and other affect scales measuring the same constructs (Table 2)*.* Results show an overall support for convergent validity of the RST-AQ. As one might expect, in Table 2 the BAS subscale is negatively related to BIS and FFFS. However, BIS and FFFS are positively related to each other. Given previous studies, this is not surprising. There is a strong debate in the literature on the differences between fear and anxiety affects (e.g., Gray, 1977; Öhman & Mineka, 2001; Sylvers et al., 2011; Corr & Cooper, 2016). For example, anxiety disorders and panic attacks (an intense and immediate response of fear) respond to different pharmacological treatments (e.g., Ravindran & Stein, 2010; American Psychiatric Association, 2013). Among other things, this allows us to think that these two affective responses are different as there are different underlying neural mechanisms. Following this discussion, it is likely that via self-report we measure anxiety-related affects as this affects manifest itself with the cognitive anticipation of a threat, whereas fear (as an immediate reaction to a present threat) is more easily measured via physiological measures. In support of this, Castro-Toledo et al. (2017) found that via physiological measures, one can capture aspects of the emotion of fear (heart rate) that are not reflected in data collected through self-report measures. Furthermore, results in Table 2, show us that our subscales are positively related to the respective subscales of the other existing measures, thus supporting convergent validity.

**Table 2:** Convergent Validity. Correlations between the BAS, BIS and FFFS subscales of RST-AQ with each other and with similar scales (High BAS is High Approach Scale; DEQ is The Descrete Emotions Questionnaire; FFFQ is The Fight, Flight and Freeze Questionnaire)

	RST-AQ BAS	RST-AQ BIS	RST-AQ FFFS
RST-AQ BAS	-		
RST-AQ BIS	-.40**	-	
RST-AQ FFFS	-.35**	.67**	-
High BAS	.75**	-.50**	-.36**
DEQ Anxiety	-.28**	.70**	.41**
DEQ Fear	-.35**	.61**	.66**
FFFQ	-.42**	.60**	.68**

**Table 3:** Relationships between traits and affects. Correlations between the BAS, BIS and FFFS subscales of RST-AQ with RST-PQ subscales (RST-PQ is Reinforcement Sensitivity Theory Personality Questionnaire)

	RST-AQ BAS	RST-AQ BIS	RST-AQ FFFS
RST-PQ BAS	.56**	-.18**	-.11
RST-PQ BIS	-.33**	.63**	.38**
RST-PQ FFFS	-.09	.38**	.29**

^**^p < .01; * *p* < .05

### RST-AQ and other instruments

#### Divergent validity

Results show at last some evidence for divergent validity with regard to the RST-AQ BIS subscale. This subscale correlates 0.45 with pessimism (Glaesmer et al., 2008), and 0.59 with RST-PQ BIS. Although small, these differences provide some evidence to support divergent validity. In the case of BAS, the evidence for divergent validity is poor, as our BAS subscale do not correlate with the RST-PQ BAS scores more than with optimism and extraversion scores (Glaesmer et al., 2008).

#### Relationships between trait and affective measures

Table 3 provides the correlations between the RST-AQ subscales and the RST-PQ scale (Corr & Cooper, 2016).

The BAS and BIS subscales of the RST-AQ moderately correlate with their matching counterparts, while correlating much lower with their non-matching counterparts (e.g., BAS trait – BIS state), thus providing evidence for convergent and divergent validity.

This is not the case with the RST-AQ FFFS subscale.

Although it hardly correlate with trait BAS, which suggests that the RST-AQ FFFS subscale possesses divergent validity in the sense that it can be separated from BAS, it does not possess that divergent validity when it comes to separating it from trait BIS. Because it even correlates better with trait BIS than with trait FFFS, albeit only slightly so, it seems to be more informative about BIS than about FFFS activation. Ironically, the RST-AQ BIS subscale is correlated slightly higher with the RST-PQ BIS scale, indicating that the RST-AQ BIS scale surpasses the RST-AQ FFFS as an indicator of FFFS.

However, these results reflect Corr and Cooper’s previous results (2016). In fact, the RST-PQ scale is also not sensitive enough in discriminating between BIS and FFFS. Obviously, as our RST-AQ scale is based on the RST-PQ, these results could be expected.

Moreover, the similarity between BIS and FFFS, both traits and affects, should be further explored in the future. It is possible that these measures do not discriminate enough not because they lack of sensitivity but due to similarities in nature between the dimensions of BIS and FFFS.

Furthermore, additional correlations were run to explore whether angry affect, as measured with the STAXI (Spielberger, 1988), is related to trait BAS sensitivity and whether a relationship exists between anger and approach-related affects. The correlation between anger and approach-related affects was negative (*r* = -0.22). On the contrary, anger was found moderately and positively correlated with BIS (*r* = 0.45) and FFFS (*r* = 0.41) affect measures. In conclusion, these findings suggest that anger and positive affects are weakly and negatively correlated. On the contrary, these results suggest that anger is related to anxiety and fear more than to positive approach-related affect it is often argued to relate to.

### Three dimensions of BAS affect

#### Correlations between the dimensions of BAS

As mentioned in the theoretical framework, we were also interested in expanding the extant research on the dimensions of approach-related affects. For this reason, we examined the relationships between the three dimensions of our BAS subscale: Joy, Interest, Activation (Egloff et al., 2003). Joy includes the items *excited, proud*, and *enthusiastic*; interest includes the items *interested, strong* and *determined*; Activation includes the items *active, alert, attentive* and *inspired*. Results showed that the three dimensions are highly and positively related to each other. However, the correlations between interest and activation (*r* = 0.73), and interest and joy (*r* = 0.71) are higher than the correlations between joy and activation *(r* = 0.65). These results are in line with our hypothesis that Interest should correlate higher with Activation and Joy than these two dimensions correlate with each other. And that is because Interest seems a more stable and dispositional dimension; whereas the other two dimensions are more changeable according to the phase of goal pursuit processes (Egloff et al., 2003). Moreover, as displayed in Table 4, we investigated the relationships between the three dimensions of our BAS subscale and the different BAS subscales of the trait RST-PQ (Reward interest, goal-drive persistence, reward reactivity, impulsivity; Corr & Cooper, 2016). In line with our expectations, results show that *activation* is positively related with both of the early stages of Reward Interest and Goal-drive persistence; *joy* is related to the late stage of Reward Responsivity and slightly but significantly with Impulsivity; *interest* with the early stages of Goal-drive persistence, Reward Interest, and moderately with the late stage of Reward Responsivity.

**Table 4:** Correlations between the three dimensions of our RST-AQ BAS and Reward Interest; Goal Drive Persistance (early stage); Reward Responsivity; Impulsivity (late stage)

	Joy	Interest	Activation
Reward Int	.44**	.48**	.48**
Goal Drive	.39**	.59**	.47**
Reward Reac	.48**	.39**	.12*
Impulsivity	.27**	.12*	.08

^**^p < .01; * *p* < .05

Furthermore, unexpectedly *joy* is positively related to the early stage of Reward Interest.

## Discussion

The RST-AQ is a newly developed scale measuring approach-related, anxious and fearful affect. The scale shows good construct validity, and its BAS and BIS subscales also show good convergent validity. Nevertheless, the scale seems not sensitive enough to discriminate well between anxious and fearful affects, as the scores of the two subscales are positively related with each other. That might be because self-report measures are more suitable for measuring anxiety rather than fear. As mentioned before, there is a strong debate about the differences between fear and anxiety (e.g., Daniel-Watanabe & Fletcher, 2022). Fear consists of an immediate physiological response to proximal threatening stimulus; whilst anxiety is an emotional response to the psychological anticipation of adverse scenarios (Gray, 1977; Öhman, et al., 2001; Sylvers et al., 2011; Corr & Cooper, 2016). Moreover, some researchers argue that fear is stimulus-specific, whilst anxiety has a diffuse nature (Gullone et al., 2000). In line with this debate, it seems logic wondering whether fear is easily measured via physiological measures, instead of self-report measures (e.g., Öhman et al., 2001; Corr & Cooper, 2016; Sylvers et al., 2011). It is likely that via self-report one measures anxiety-related affect associated with the cognitive anticipation of a threat; whereas fear (as an immediate reaction to a present threat) is more easily measured via physiological measures (e.g., Castro-Toledo et al., 2017). In our survey, it is possible that participants were reporting anxiety-related affects also when they were asked about fearful affects. Further studies are needed to better disentangle these affects.

Correlations between trait and affective measures also provided evidence for convergent validity. The BIS subscale of the RST-AQ was moderately and positively related to trait BIS; the BAS subscale of the RST-AQ was related positively but slightly to trait BAS. Only the FFFS subscale of the RST-AQ failed the tests for convergent and divergent validity. Furthermore, it was also explored whether anger was related to approach, fearful or anxious related affects. As shown in our data, angry affect is related to anxious and fearful affect (e.g., Khoshfetrat et al., 2022; McGarry & Shortland, 2023). This suggests that anger (together with anxiety and fear) is an affect experienced as indicator of BIS or FFFS activation. Before drawing strong conclusions about the relationships between anxiety, positive affect and anger, and their relationship to approach motivation, we should explore some possible explanations for our finding that anger was positively related to BIS and FFFS yet negatively related to BAS. One explanation is that people’s cognitions about themselves and others influence affective self-reports. In fact self-report measures are sensitive to social desirability. This may be particularly true for anger because anger is not always a socially accepted emotion (e.g., Ramtahal, 2017) and this might discourage people from reporting their experience of anger. This would suggest to explore anger further in an experimental design that comprises prevention and detection of social desirability methods or indirect questioning, which are also valid measures employed to reduce social desirability biases (Nederhof, 1985; Fisher and Katz, 1993; Weydmann et al., 2020). A second possible explanation might be that a person who scores higher on positive affect might experience anger but report the experience as feeling energized. This is because physiological arousal occurs before the cognitive process of labeling of the emotions occurs. In particular, Schacter-Singer Theory suggests that similar physiological responses can be labeled as different emotions, depending on the internal and external context this activation occurs in (Schachter & Singer, 1962; Schachter, 1964, 1980). Participants might mislabel anger as feeling energized. Feeling energized is one of the approach-related affects measured by BAS state scales. According to Schachter-Singer Theory, having already reported experiencing positive affect would function as a context to interpret the physiological activation of anger as a symptom of positive affect. On the contrary, if labeling the physiological state as anger, participants might experience cognitive dissonance (Festinger, 1980). This may happen if one person might report experiencing positive affect, and feel discomfort when having to report the experience of anger at the same time. In other words, participants might label their own physiological activation differently depending on their general affective experience. One person who scores higher in positive affects might label this physiological activation “feeling energized” as feeling energized is one of the approach related affects measured by BAS state scales; whilst another person who scores higher in BIS related affects might call the same physiological activation “anger”. In order to explore further these relationships between anger and approach-related affects/traits, physiological measures are needed.

### Limitations and future avenues

This line of thoughts brings us to the limitations of our study. First, our study is a self-report survey. Self-report measures, as mentioned above, are influenced by social desirability (Ramtahal, 2017). Second, our study would benefit of an integration with an experimental design that can measure the physiological responses as well as the cognitive process of labeling these physiological activations. Third, in the present study participants are asked to indicate *how they generally feel*, instead of *how they feel right now.* Asking *how they feel right now*, might be more sensitive in capturing the transitory affective state of people.

Furthermore, although the sample size of our study was determined a-priori using an online calculator (https://www.danielsoper.com/statcalc/calculator.aspx?id=89); future studies exploring BIS, BAS and FFFS states would benefit of recruiting more participants for a bigger sample.

However, in spite of these limitations, the RST-AQ measure is, as far as we know, the first instrument that provides researchers with a scale to measures approach, anxiety and fearful related affects.

### Research implications

The manuscript has at least two research implications. First, it serves researchers investigating the field of affects with the first validated instruments on the approach, fearful, anxious related affects. Especially given the number of studies revealing the importance of the dimensions of BIS, BAS and FFFS in the emotional regulation processes and emotional intelligence (e.g., Tull et al., 2010; Baumann et al., 2007). Second, it contributes to the extant knowledge on affects, further exploring the relationships between BAS, BIS, FFFS-related affects, the subdimensions of BAS, and the relationships between anger and BAS, BIS and FFFS states.

## Conclusions

In this paper, it is reported the validation of a newly developed self-report measures. The RST-AQ provides researchers with one comprehensive and simplified instruments for BIS, BAS and FFFS states. This scale is created using different existing self-report scales on affects and combining them in our newly developed affect self-report scale.

Furthermore, we investigated whether trait measures of the three systems are related to anxious, approach-related and fearful affects. Our aim was to extend the extant literature of BIS, BAS and FFFS related affects, providing researchers with one self-report measure to investigate this topic further. This is extremely important because RST, besides being a theory of personality traits and motivational states, is a theory of emotional states of people. Moreover, so far there was no existing self-report measure researchers could use to assess affects. Hopefully, this study might lay the groundwork for future studies in the field of RST emotional states of people.

However, BIS, BAS, and FFFS states should be explored further using both self-report measures and physiological measures in order to fully disentangle and understand these constructs.

## Data Availability

The datasets generated and analysed during the current study are available from the corresponding author on reasonable request.

## Appendix

### RST-AQ Measure

*Now we would like** you to tell us how you generally feel. The following words describe different feelings and sensations. Read each word and then write the intensity in the scale next to each word. You have the option of choosing from seven gradations (Does not apply to me at all* = *0; Does apply to me completely* = *7). Please indicate how you generally feel.*

BAS affects

- active
- alert
- determined
- enthusiastic
- excited
- inspired
- interested
- proud
- strong

BIS affects

- afraid
- scared
- frightened
- nervous
- jittery
- shaky
- worried
- inhibited

FFFS affects

- horrified
- terrified
- petrified
- startled
- shocked

## References

[CR1] Agroskin D, Jonas E, Klackl J, Prentice M (2016). Inhibition underlies the effect of high need for closure on cultural closed-mindedness under mortality salience. Frontiers in Psychology.

[CR2] Alloy LB, Abramson LY, Walshaw PD, Gerstein RK, Keyser JD, Whitehouse WG, Harmon-Jones E (2009). Behavioral approach system (BAS)–relevant cognitive styles and bipolar spectrum disorders: Concurrent and prospective associations. Journal of Abnormal Psychology.

[CR3] American Psychiatric Association (2013). Diagnostic and statistical manual of mental disorders - (5th ed.).

[CR4] Bacon AM, Corr PJ (2017). Motivating emotional intelligence: A reinforcement sensitivity theory (RST) perspective. Motivation and EmotIon.

[CR5] Baumann N, Kaschel R, Kuhl J (2007). Affect sensitivity and affect regulation in dealing with positive and negative affect. Journal of Research in Personality.

[CR6] Bijttebier P, Beck I, Claes L, Vandereycken W (2009). Gray's Reinforcement Sensitivity Theory as a framework for research on personality–psychopathology associations. Clinical Psychology Review.

[CR7] Blanchard DC, Griebel G, Blanchard RJ (2001). Mouse defensive behaviors: pharmacological and behavioral assays for anxiety and panic. Neuroscience & Biobehavioral Reviews.

[CR8] Boateng GO, Neilands TB, Frongillo EA, Melgar-Quiñonez HR, Young SL (2018). Best practices for developing and validating scales for health, social, and behavioral research: A primer. Frontiers in Public Health.

[CR9] Bowers D, Heilman KM (1980). Pseudoneglect: Effects of hemispace on a tactile line bisection task. Neuropsychologia.

[CR10] Bryan CJ, Kyron M, Page AC (2022). BIS sensitivity, BAS sensitivity, and recent suicide attempts. Personality and Individual Differences.

[CR11] Carver CS (2004). Negative affects deriving from the behavioral approach system. Emotion.

[CR12] Carver CS, White TL (1994). Behavioral inhibition, behavioral activation, and affective responses to impending reward and punishment: The BIS/BAS scales. Journal of Personality and Social Psychology.

[CR13] Castro-Toledo FJ, Perea-García JO, Bautista-Ortuño R (2017). Influence of environmental variables on fear of crime: Comparing self-report data with physiological measures in an experimental design. Journal of Experimental Criminology.

[CR14] Chat IKY, Dunning EE, Bart CP, Carroll AL, Grehl MM, Damme KS, Alloy LB (2022). The interplay between reward-relevant life events and trait reward sensitivity in neural responses to reward cues.&nbsp;Clinical. Psychological Science.

[CR15] Cooper A, Gomez R, Buck E (2008). The relationships between the BIS and BAS, anger and responses to anger. Personality and Individual Differences.

[CR16] Cooper AJ, Stirling S, Dawe S, Pugnaghi G, Corr PJ (2017). The reinforcement sensitivity theory of personality in children: A new questionnaire. Personality and Individual Differences.

[CR17] Corr PJ (2004). Reinforcement sensitivity theory and personality. Neuroscience & Biobehavioral Reviews.

[CR18] Corr PJ, Cooper AJ (2016). The Reinforcement Sensitivity Theory of Personality Questionnaire (RST-PQ): Development and validation. Psychological Assessment.

[CR19] Corr PJ, McNaughton N (2012). Neuroscience and approach/avoidance personality traits: A two stage (valuation–motivation) approach. Neuroscience & Biobehavioral Reviews.

[CR20] Crocq, M. (2017). The history of generalized anxiety disorder as a diagnostic category. *Dialogues in Clinical Neuroscience, 19*(2), 107–116. 10.31887/DCNS.2017.19.2/macrocq PMC5573555 28867935

[CR21] Daniel-Watanabe L, Fletcher PC (2022). Are fear and anxiety truly distinct?. Biological Psychiatry Global Open Science.

[CR22] De Beni R, Moè A (2003). Imagery and rehearsal as study strategies for written or orally presented passages. Psychonomic Bulletin & Review.

[CR23] Egloff B, Schmukle SC, Burns LR, Kohlmann C-W, Hock M (2003). Facets of dynamic positive affect: Differentiating joy, interest, and activation in the positive and negative affect schedule (PANAS). Journal of Personality and Social Psychology.

[CR24] Espinoza Oyarce DA, Burns R, Butterworth P, Cherbuin N (2021). Bridging classical and revised reinforcement sensitivity theory research: A longitudinal analysis of a large population study. Frontiers in Psychology.

[CR25] Festinger L (1980). Retrospections on social psychology.

[CR26] Field A (2009). Discovering statistics using SPSS.

[CR27] Fischer MH (1994). Less attention and more perception in cued line bisection. Brain and Cognition.

[CR28] Fisher R, Katz JE (1993). Social desirability bias and the validity of indirect questioning. Journal of Consumer Research.

[CR29] Fowles DC (1987). Application of a behavioral theory of motivation to the concepts of anxiety and impulsivity. Journal of Research in Personality.

[CR30] Franken IH (2002). Behavioral approach system (BAS) sensitivity predicts alcohol craving. Personality and Individual Differences.

[CR31] Glaesmer H, Hoyer J, Klotsche J, Herzberg PY (2008). zum dispositionellen Optimismus und Pessimismus [the German version of the life orientation test (LOT-R) on dispositional optimism and pessimism]. Zeitschrift Für Gesundheitspsychologie.

[CR32] Glaesmer H, Rief W, Martin A, Mewes R, Brähler E, Zenger M, Hinz A (2012). Psychometric properties and population based norms of the Life Orientation Test Revised (LOT-R). British Journal of Health Psychology.

[CR33] Gray, J. A. (1977). Drug effects on fear and frustration: Possible limbic site of action of minor tranquilizers. *Drugs, Neurotransmitters, and Behavior*, 433–529.

[CR34] Greenaway KH, Storrs KR, Philipp MC, Louis WR, Hornsey MJ, Vohs KD (2015). Loss of control stimulates approach motivation. Journal of Experimental Social Psychology.

[CR35] Gross JJ (2013). Emotion regulation: taking stock and moving forward. Emotion.

[CR36] Guinote A (2017). How Power Affects People: Activating, Wanting, and Goal Seeking. Annual Review of Psychology..

[CR37] Gullone E, King NJ, Ollendick TH (2000). The development and psychometric evaluation of the Fear Experiences Questionnaire: An attempt to disentangle the fear and anxiety constructs. Clinical Psychology & Psychotherapy: An International Journal of Theory & Practice.

[CR38] Harmon-Jones C, Bastian B, Harmon-Jones E (2016). Detecting transient emotional responses with improved self-report measures and instruction. Emotion.

[CR39] Hatin B, Tottenham LS (2016). What’s in a line? Verbal, facial, and emotional influences on the line bisection task. Laterality: Asymmetries of Body, Brain and Cognition.

[CR40] Heidari P, Nemattavousi M (2021). behavioral inhibition/activation systems and self-esteem with depression: The mediating role of social anxiety. Journal of Rational-Emotive & Cognitive-Behavior Therapy.

[CR41] Henson RK, Roberts JK (2006). Use of exploratory factor analysis in published research: Common errors and some comment on improved practice. Educational and Psychological Measurement.

[CR42] Heponiemi T, Keltikangas-Järvinen L, Puttonen S, Ravaja N (2003). BIS/BAS sensitivity and self-rated affects during experimentally induced stress. Personality and Individual Differences.

[CR43] Hu LT, Bentler PM (1999). Cutoff criteria for fit indexes in covariance structure analysis: Conventional criteria versus new alternatives. Structural Equation Modeling: A Multidisciplinary Journal.

[CR44] Hu W, Zhao X, Liu Y, Ren Y, Wei Z, Tang Z, Yang J (2022). Reward sensitivity modulates the brain reward pathway in stress resilience via the inherent neuroendocrine system. Neurobiology of Stress.

[CR45] Hundt NE, Nelson-Gray RO, Kimbrel NA, Mitchell JT, Kwapil TR (2007). The interaction of reinforcement sensitivity and life events in the prediction of anhedonic depression and mixed anxiety-depression symptoms. Personality and Individual Differences.

[CR46] Kajdzik, M., & Moroń, M. (2023). Signaling high sensitivity to influence others: Initial evidence for the roles of reinforcement sensitivity, sensory processing sensitivity, and the dark triad. *Psychological Reports.*10.1177/00332941231152387 10.1177/00332941231152387 36645280

[CR47] Kaynak, H., Turan, A., Demir, Y. (2022). Locus of control as a mediator of the relationships between motivational systems and trait anxiety. *Psychological Reports. *10.1177/00332941221139707. 10.1177/00332941221139707 36377649

[CR48] Khoshfetrat A, Scully D, Fassbender C (2022). Effects of behavioral inhibition/activation systems on anger rumination and anger expression through difficulty in emotion Regulation. Personality and Individual Differences.

[CR49] Kidd C, Loxton NJ (2021). A narrative review of reward sensitivity, rash impulsivity, and food addiction in adolescents. Progress in Neuro-Psychopharmacology and Biological Psychiatry.

[CR50] Kline TJ (2005). Psychological testing: A practical approach to design and evaluation.

[CR51] Lee BH, Kim M, Kang SJ, Park KC, Kim EJ, Adair JC, Na DL (2004). Pseudoneglect in solid-line versus character-line bisection tasks: A test for attention dominance theory. Cognitive and Behavioral Neurology.

[CR52] Leggett NC, Thomas NA, Nicholls MER (2016). End of the line: Line bisection, an unreliable measure of approach and avoidance motivation. Cognition and Emotion.

[CR53] Li Y, Xu Y, Chen Z (2015). Effects of the behavioral inhibition system (BIS), behavioral activation system (BAS), and emotion regulation on depression: A one-year follow-up study in Chinese adolescents. Psychiatry Research.

[CR54] Maack DJ, Buchanan E, Young J (2015). Development and psychometric investigation of an inventory to assess fight, flight, and freeze tendencies: the fight, flight freeze questionnaire. Cognitive Behaviour Therapy.

[CR55] McFarland BR, Shankman SA, Tenke CE, Bruder GE, Klein DN (2006). Behavioral activation system deficits predict the six-month course of depression. Journal of Affective Disorders.

[CR56] McGarry P, Shortland N (2023). Anxious activism: The role of behavioral inhibition system in the radicalization process. Computers in Human Behavior.

[CR57] McGregor I, Nash K, Mann N, Phills CE (2010). Anxious uncertainty and reactive approach motivation (RAM). Journal of Personality and Social Psychology.

[CR58] McNaughton N, Corr PJ (2004). A two dimensional neuropsychology of defense: Fear/anxiety and defensive distance. Neuroscience & Biobehavioral Reviews.

[CR59] McNaughton N, Corr PJ (2008). The neuropsychology of fear and anxiety: A foundation for Reinforcement Sensitivity Theory.

[CR60] McNaughton N, Gray JA (2000). Anxiolytic action on the behavioural inhibition system implies multiple types of arousal contribute to anxiety. Journal of Affective Disorders.

[CR61] Merchán-Clavellino A, Alameda-Bailén JR, Zayas García A, Guil R (2019). Mediating effect of trait emotional intelligence between the behavioral activation system (BAS)/behavioral inhibition system (BIS) and positive and negative affect. Frontiers in Psychology.

[CR62] Milner AD, Brechmann M, Pagliarini L (1992). To halve and to halve not: an analysis of line bisection judgements in normal subjects. Neuropsychologia.

[CR63] Moè A, Pazzaglia F, Ronconi L (2010). When being able is not enough. The combined value of positive affect and self-efficacy for job satisfaction in teaching. Teaching and Teacher Education.

[CR64] Moè A, Hausmann M, Hirnstein M (2021). Gender stereotypes and incremental beliefs in STEM and non-STEM students in three countries: Relationships with performance in cognitive tasks. Psychological Research.

[CR65] Moncel C, Osmont A, Pavani JB, Pichot N, Dauvier B (2023). Development and validation of hierarchically structured questionnaire of approach and avoidance motivation: Validation of the hierarchical reinforcement sensitivity theory of personality questionnaire. Personality and Individual Differences.

[CR66] Nash K, McGregor I, Prentice M (2011). Threat and defense as goal regulation: From implicit goal conflict to anxious uncertainty, reactive approach motivation, and ideological extremism. Journal of Personality and Social Psychology.

[CR67] Nederhof AJ (1985). Methods of coping with social desirability bias: A review. European Journal of Social Psychology.

[CR68] Öhman A, Mineka S (2001). Fears, phobias, and preparedness: Toward an evolved module of fear and fear learning. Psychological Review.

[CR69] Perusini JN, Fanselow MS (2015). Neurobehavioral perspectives on the distinction between fear and anxiety. Learning Memory.

[CR70] Pett MA, Lackey NR, Sullivan JJ (2003). Making sense of factor analysis: The use of factor analysis for instrument development in health care research.

[CR71] Pugnaghi G, Cooper A, Ettinger U, Corr PJ (2018). The psychometric properties of the German language reinforcement sensitivity theory-personality questionnaire (RST-PQ). Journal of Individual Differences.

[CR72] Pugnaghi G, Memmert D, Kreitz C (2019). Examining effects of preconscious mere exposure: An inattentional blindness approach. Consciousness and Cognition.

[CR73] Pulido MA, Brown F, Cortés R, Salame M (2023). Reinforcement sensitivity theory may predict COVID-19 infection outcome and vulnerability. Personality and Individual Differences.

[CR74] Ramtahal NA (2017). Anger self-report and social desirability bias: Am I angry or am I biased?.

[CR75] Ravindran LN, Stein MB (2010). The pharmacologic treatment of anxiety disorders: A review of progress. The Journal of Clinical Psychiatry.

[CR76] Schachter S (1964). The interaction of cognitive and physiological determinants of emotional state. Advances in Experimental Social Psychology.

[CR77] Schachter S, Singer J (1962). Cognitive, social, and physiological determinants of emotional state. Psychological Review.

[CR78] Schmeichel BJ, Harmon-Jones C, Harmon-Jones E (2010). Exercising self-control increases approach motivation. Journal of Personality and Social Psychology.

[CR79] Spielberger CD, Krasner SS, Solomon EP, Janisse MP (1988). The experience, expression and control of anger. Health psychology: Individual differ[1]ences and stress.

[CR80] Steimer, T. (2022). The biology of fear-and anxiety-related behaviors. *Dialogues in Clinical* Neuroscience. 10.31887/DCNS.2002.4.3/tsteimer PMC3181681 22033741

[CR81] Stevens JP (2009). Applied multivariate statistics for the social sciences.

[CR82] Strand N (2023). Gender/sex differences in enjoyment of threat-related images and considerations of approach-avoidance and resilience traits.

[CR83] Sylvers P, Lilienfeld SO, LaPrairie JL (2011). Differences between trait fear and trait anxiety: Implications for psychopathology. Clinical Psychology Review.

[CR84] Torrubia R, Avila C, Moltó J, Caseras X (2001). The Sensitivity to Punishment and Sensitivity to Reward Questionnaire (SPSRQ) as a measure of Gray's anxiety and impulsivity dimensions. Personality and Individual Differences.

[CR85] Tull MT, Gratz KL, Latzman RD, Kimbrel NA, Lejuez CW (2010). Reinforcement sensitivity theory and emotion regulation difficulties: A multimodal investigation. Personality and Individual Differences.

[CR86] Vagg PR, Spielberger CD (1979). State trait anger expression inventory interpretive report (STAXI-2: IR). Psychological Assessment Resources Inc.

[CR87] Watson D, Clark LA (1999). The PANAS-X manual for the positive and negative affect schedule expanded form.

[CR88] Weydmann G, Hauck Filho N, Bizarro L (2020). Acquiescent responding can distort the factor structure of the BIS/BAS scales. Personality and Individual Differences.

[CR89] Wilson DR, Loxton NJ, O’Donovan A (2021). From BIS to binge: The role of negative affect in the pathway between personality and binge eating. Elsevier Eating Behaviors.

[CR90] Włodarska KA, Zyskowska E, Terebus MK, Rogoza R (2021). The dark triad and BIS/BAS: A meta-analysis. Current Psychology.

[CR91] Wu R, Huang J, Ying J, Gao Q, Guo J, You J (2021). Behavioral inhibition/approach systems and adolescent nonsuicidal self-injury: The chain mediating effects of difficulty in emotion regulation and depression. Personality and Individual Differences.

[CR92] Wytykowska A, Fajkowska M, Domaradzka E (2021). BIS-dependent cognitive strategies mediate the relationship between BIS and positive, negative affect. Personality and Individual Differences.

